# Particle Filter with Novel Nonlinear Error Model for Miniature Gyroscope-Based Measurement While Drilling Navigation

**DOI:** 10.3390/s16030371

**Published:** 2016-03-15

**Authors:** Tao Li, Gannan Yuan, Wang Li

**Affiliations:** Marine Navigation Research Institute, College of Automation, Harbin Engineering University, No. 145 Nan Tong Street, Harbin 150001, China; yuangannan@hrbeu.edu.cn (G.Y.); liwang@hrbeu.edu.cn (W.L.)

**Keywords:** NNEM, MGWD, PF, multilateral horizontal drilling, quasi-stationary condition, MEMS

## Abstract

The derivation of a conventional error model for the miniature gyroscope-based measurement while drilling (MGWD) system is based on the assumption that the errors of attitude are small enough so that the direction cosine matrix (DCM) can be approximated or simplified by the errors of small-angle attitude. However, the simplification of the DCM would introduce errors to the navigation solutions of the MGWD system if the initial alignment cannot provide precise attitude, especially for the low-cost microelectromechanical system (MEMS) sensors operated in harsh multilateral horizontal downhole drilling environments. This paper proposes a novel nonlinear error model (NNEM) by the introduction of the error of DCM, and the NNEM can reduce the propagated errors under large-angle attitude error conditions. The zero velocity and zero position are the reference points and the innovations in the states estimation of particle filter (PF) and Kalman filter (KF). The experimental results illustrate that the performance of PF is better than KF and the PF with NNEM can effectively restrain the errors of system states, especially for the azimuth, velocity, and height in the quasi-stationary condition.

## 1. Introduction

Horizontal drilling (HD), especially reentry multilateral horizontal well drilling (RMHWD), receives considerable attention because it provides great economic value in the oil and gas industry. The RMHWD technology was originally proposed in an industry consortium of operators and service companies in 1997 [1] and RMHWD can significantly increase the production of oil and gas by extending the contact areas between oil and gas reservoirs and the well drilling equipment and by revisiting the existing wellbores [2]. RMHWD is also readily available under challenging drilling sites such as offshore zones, mountain areas, and downtown centers [3]. The branches of the drill pipe in RMHWD radiate from the main wellbore to the targeted reservoir sections [4]. The high pressure waterjet drilling technology promotes the development of RMHWD since the conventional large rotary drill bit can be replaced by a small-diameter waterjet drill bit. The conventional measurement-while-drilling (MWD) tool combines three-axis gyroscopes and three-axis magnetometers to provide the attitude (tool face, inclination, and azimuth) of the drill bit. However, the performance of the MWD tools would deteriorate under the strong magnetic interference caused by the following factors: (1) flow of currents in the atmosphere and moving of solar wind [5]; (2) existing drilling fluid and debris [6,7]; (3) ferromagnetic deposits in the proximity of bottom hole assembly (BHA) [8]. Non-magnetic drill collars can effectively eliminate the magnetic disturbance; however, the high cost, high weight, and weak fracture drill collars fail to be widely used.

Alternatively, gyroscope-based MWD (GWD) tools can continuously provide the attitude, velocity, and position of the drill bit regardless of the strong magnetic interferences. GWD tools with pure strapdown inertial navigation algorithm were proposed in [9,10] and the tools can save considerable rig time and remove the blind during the kickoff [11]. The size of the fiber optic gyroscope or the laser optic gyroscope limits their application to the surveying of downhole drilling. The emergence of a low-cost, small-size, and high-performance MEMS sensor brings the gyroscopes to the small-diameter RMHWD. The MGWD device with two-axis gyroscopes and three-axis accelerometers in the application of small-diameter space (less than 24 millimeters) was proposed in [12], in which the angular rate (Y-axis) of MGWD tools is calculated by the accelerometers in the horizontal plane when the drilling equipment is under quasi-stationary conditions. The MGWD system can provide high-precision navigation solutions in short-term [13] and can perform real-time data transmission between the MGWD tools and the ground operation center. However, the integration calculation within resolving the mechanization of navigation solutions accumulates large errors because of biases of gyroscopes and accelerometers. The errors of inertial sensors are composed of two parts: deterministic errors and stochastic errors [14]. The deterministic errors can partially be reduced by the calibration approach. Therefore, the residual of deterministic errors and the stochastic errors are main error sources of inertial sensors. To sum up, the problems should be solved for MGWD system are the elimination of the residual of deterministic errors and stochastic errors.

The stochastic errors of inertial sensors can be modeled by the first-order Gaussian–Markov (GM) model or the high-order autoregressive (AR) model [15]. The Levenberg–Marquardt iterative least squares can fit the nonlinear parametric model of stochastic errors as well [16]. The traditional error models of the MGWD system, such as the Phi-angle or Psi angle model, are based on the assumption that the attitude errors are small enough that the DCM can be simplified by the small-angle attitude errors. The linear modified error model for large heading uncertainty was proposed in [17] and the model of moving alignment with large errors was put forward in [18]. In the application of the Kalman filter (KF), both error models neglect the high-order error terms that may introduce errors when the attitude errors are large. In addition, the system noises and the observation noises are not Gaussian for MGWD system in sophisticated downhole drilling environments (high temperature and high pressure). Therefore, the KF is suboptimal compared with the particle filter (PF), which is the recursive approximation of the posterior probability density function (pdf) with particles and weights [19]. The conventional in-drilling alignment (IDA) approaches include the zero velocity update (ZUPT) alignment, velocity matching alignment, rotary modulation alignment, *etc.* The ZUPT alignment can limit the position errors up to 40 m during a 90-min experiment by using a Litton LTN90-100 inertial measurement unit (IMU) [20]. The velocity matching alignment merges velocity from global positioning system (GPS) or other velocity sensors to the filtering for compensating the errors [21]. An IDA approach was introduced to restrict the azimuth error of the drill bit in [22,23]; the azimuth error was reduced 25 times smaller than the conventional magnetometer based MWD tools. The rotary-in-drilling alignment (RIDA) was proposed in terms of the theory of rotary modulation alignment in an inertial navigation system (INS) to minimize the dynamic position errors and the azimuth error of the MWD system [24,25]. However, GPS signals cannot reach to a deep downhole and the size of IDA or RIDA is beyond the dimension requirements of the RMHWD.

This paper proposes a NNEM for the MGWD system under large-angle attitude error conditions. The error matrix of DCM is introduced to represent the attitude errors. Because of the nonlinearity of the system state space model (SSM), the PF is selected as the state estimation approach. The body frame is the right, forward, and up direction of the MGWD system and the navigation frame is the north, east, and down direction in this paper.

In Section 2, the NNEM of the MGWD system is proposed. Section 3 and Section 4 introduce the recursive Bayesian estimation theory and PF algorithm, respectively. Results and experiments are explained in Section 5. Eventually, conclusions are presented in Section 6.

## 2. NNEM of the MGWD System

The error model of the strapdown inertial navigation system (SINS), based on the discrete time differential equation and the error model, plays a fundamental role in the estimation of system states. Conventional linear error models (LEM) of SINS, such as the Phi-angle model, the Psi-angle model [26,27], and the quaternion error model [28] are widely used in aerospace and marine navigation. The Phi-angle error model analyzes the SINS in the true navigation frame while the Psi-angle model is performed in the computer frame. In this paper, the Psi-angle model is selected for the states estimation of KF while the error model in [29] is used as the NEM for the states estimation of the PF.

The derivation of a linear model by the perturbation method alone can be implemented in light of the assumption that the attitude errors are small enough. The initial alignment of the MGWD system cannot provide an accurate attitude because of the low performance of the MEMS sensors and the sophisticated downhole drilling environments. Therefore, this paper proposes an accurate NNEM of the MGWD system under large-angle attitude error conditions.

The key variable for the derivation of the SINS error model is the rotation vector, which is the eigenvector of the DCM associated with eigenvalue 1. The eigenvector of the DCM can be expressed as follows [30,31]:

(1) $$(R_{b}^{n}-I)\Phi =0$$

where $R_{b}^{n}$ is the DCM from the body frame to the navigation frame, Φ is the rotation vector, and I is a 3×3 identity matrix. The rotation vector is proposed in [32], and the relationship between rotation vector and DCM can be expressed as:

(2) $$R_{b}^{n}=I+\frac{\sin\Phi }{\Phi }\left(\Phi \times \right)+\frac{1-\text{cos}\Phi }{\Phi^{2}}{\left(\Phi \times \right)}^{2}$$

where (Φ×) is the skew symmetric matrix of Φ, *i.e.,*:

(3) $$\left(\Phi \times \right)=\left[\begin{array}{ccc} 0 & -\Phi_{z} & \Phi_{y}\\ \Phi_{z} & 0 & -\Phi_{x}\\ -\Phi_{y} & \Phi_{x} & 0 \end{array}\right]$$

where $\Phi_{x},\;\Phi_{y}$, and $\Phi_{z}$ are the components of Φ projected in X-axis, Y-axis, and Z-axis.

The magnitude of Φ equals:

(4) $$\Phi ={\left(\Phi^{T}\Phi \right)}^{1/2}$$

So far, the problem of seeking an attitude solution equates to seeking a solution for the rotation vector. The derivation of the kinematic equation of rotation vector was reviewed in [33], and the conventional differential equation of rotation vector was given by [34]:

(5) $$\dot{\Phi }=\left(I+\frac{1}{2}\left(\Phi \times \right)+\frac{1}{\Phi^{2}}\left(1-\frac{\Phi \sin\Phi }{2\left(1-\text{cos}\Phi \right)}\right){\left(\Phi \times \right)}^{2}\right)\omega_{\text{nb}}^{b}$$

where $\omega_{\text{nb}}^{b}$ is the angular rate of the body frame with respect to the navigation frame resolved in the body frame.

This section reviews the nonlinear error model of MGWD according to [27], in which the attitude error model is derived according to the linear quaternion error model. The attitude errors are defined in terms of the rotation vector:

(6) Φ=(δϕ,δθ,δψ)

where δϕ, δθ, and δψ are the roll error, pitch error, and azimuth error. The NNEM of the attitude can be expressed as follows:

(7) $$\dot{\Phi }={\left(I-c_{n}{\Phi \Phi }^{T}\right)}^{-1}\left(-\omega_{\text{in}}^{n}\times \Phi -\frac{1}{2s_{n}}\left(R_{b}^{n}\omega_{\text{ib}}^{b}-{\delta \omega }_{\text{in}}^{n}\right)\right)$$

where $c_{n}$ and $s_{n}$ are defined parameters that can be expressed as:

(8) $$c_{n}=\cos\left(\frac{\Phi_{n}}{2}\right)-1$$

(9) $$s_{n}=\frac{1}{\Phi_{n}}\sin\left(\frac{\Phi_{n}}{2}\right)$$

(10) $$\Phi_{n}=\sqrt{\delta \phi^{2}+{\delta \theta }^{2}+{\delta \psi }^{2}}$$

where $\omega_{\text{ib}}^{b}$ is the angular rate obtained from the gyroscope, $\omega_{\text{in}}^{n}$ is the angular rate of the navigation frame with respect to the inertial frame resolved in the navigation frame, and $\omega_{\text{in}}^{n}$ can be expressed as:

$$\omega_{\text{in}}^{n}=\omega_{\text{ie}}^{n}+\omega_{\text{en}}^{n}$$

where $\omega_{\text{ie}}^{n}$ is the Earth rotation rate in the navigation frame and $\omega_{\text{ie}}^{n}$ can be written as follows:

(11) $$\omega_{\text{ie}}^{n}={\left[\begin{array}{ccc} \omega_{\text{ie}}\text{cos}\varphi & 0 & -\omega_{\text{ie}}\sin\varphi \end{array}\right]}^{T}$$

where $\omega_{\text{ie}}$ is the Earth rotation rate $7.292115\times 10^{-5}\text{rad}/s\approx 15.041067{}^{\circ}/h$; φ is the latitude; $\omega_{\text{en}}^{n}$ is the angular rate of the navigation frame with respect to the Earth frame resolved in the navigation frame; and $\omega_{\text{en}}^{n}$ can be written as:

(12) $$\omega_{\text{en}}^{n}={\left[\begin{array}{ccc} \frac{V_{E}}{R_{N}+h} & -\frac{V_{N}}{R_{M}+h} & -\frac{V_{E}\text{tan}\varphi }{R_{N}+h} \end{array}\right]}^{T}$$

where $V_{E}\text{ and }V_{N}$ represent the east velocity and north velocity, respectively, in the navigation frame; h is the ellipsoidal height; $R_{N}\text{ and }R_{M}$ are the radii of curvature in the meridian and the prime vertical, respectively; and $R_{N},R_{M}$ can be expressed as follows [35]:

(13) $$R_{N}=\frac{R}{{\left(1-e^{2}\sin^{2}\varphi \right)}^{1/2}}$$

(14) $$R_{M}=\frac{R\left(1-e^{2}\right)}{{\left(1-e^{2}\sin^{2}\varphi \right)}^{3/2}}$$

where R, e are the semi-major axis and linear eccentricity of the reference ellipsoid, respectively, in which R=6378137 m and e=0.0818191908426.

The output errors of accelerometers and gyroscopes (${\delta f}_{\text{ib}}^{b}$ and ${\delta \omega }_{\text{ib}}^{b}$) can be expressed as [36]:

(15) $${\delta \omega }_{\text{ib}}^{b}=\left[\begin{array}{ccc} -{\Delta \text{SF}}_{x,g} & {\Delta \delta }_{\text{xz},g} & -{\Delta \delta }_{\text{xy},g}\\ -{\Delta \delta }_{\text{yz},g} & -{\Delta \mathrm{SF}}_{y,g} & {\Delta \delta }_{\text{yx},g}\\ {\Delta \delta }_{\text{zy},g} & -{\Delta \delta }_{\text{zx},g} & -{\Delta \mathrm{SF}}_{z,g} \end{array}\right]\omega_{\text{ib}}^{b}+\left[\begin{array}{c} \epsilon_{x,g}\\ \epsilon_{y,g}\\ \epsilon_{z,g} \end{array}\right]$$

(16) $${\delta f}_{\text{ib}}^{b}=\left[\begin{array}{ccc} -{\Delta \text{SF}}_{x,a} & {\Delta \delta }_{\text{xz},a} & -{\Delta \delta }_{\text{xy},a}\\ -{\Delta \delta }_{\text{yz},a} & -{\Delta \text{SF}}_{y,a} & {\Delta \delta }_{\text{yx},a}\\ {\Delta \delta }_{\text{zy},a} & -{\Delta \delta }_{\text{zx},a} & -{\Delta \mathrm{SF}}_{z,a} \end{array}\right]f_{\text{ib}}^{b}+\left[\begin{array}{c} \epsilon_{x,a}\\ \epsilon_{y,a}\\ \epsilon_{z,a} \end{array}\right]$$

where ${\Delta \mathrm{SF}}_{x,g}$ and ${\Delta \mathrm{SF}}_{z,g}$ are the scale factor errors of X-axis gyroscope and Z-axis gyroscope. ${\Delta \mathrm{SF}}_{y,g}$ is defined as the scale factor error of a virtual gyroscope along the Y-axis. ${\Delta \delta }_{\text{xy},g},\;{\Delta \delta }_{\text{xz},g},\;{\Delta \delta }_{\text{yz},g},\;{\Delta \delta }_{\text{yx},g},\;{\Delta \delta }_{\text{zy},g},$ and ${\Delta \delta }_{\text{zx},g}$ are non-orthogonal gyroscope errors along the X-axis, Y-axis, and Z-axis. ${\Delta \mathrm{SF}}_{x,a},\;{\Delta \mathrm{SF}}_{y,a},$ and ${\Delta \mathrm{SF}}_{z,a}$ are the scale factor errors of the X-axis accelerometer, Y-axis accelerometer, and Z-axis accelerometer, respectively. ${\Delta \delta }_{\text{xy},a},\;{\Delta \delta }_{\text{xz},a},\;{\Delta \delta }_{\text{yz},a},\;{\Delta \delta }_{\text{yx},a},\;{\Delta \delta }_{\text{zy},a},\text{ and }{\Delta \delta }_{\text{zx},g}$ are non-orthogonal acceleration errors along the X-axis, Y-axis, and Z-axis. $\epsilon_{x,g},\epsilon_{y,g},\epsilon_{z,g}$ and $\epsilon_{x,a},\epsilon_{y,a},\epsilon_{z,a}$ are the angular drift errors of gyroscopes and drift errors of accelerometers along the X-axis, Y-axis, and Z-axis, respectively.

Through differentiating the Equations (11) and (12), ${\delta \omega }_{\text{in}}^{n}$ can be written as [37]:

(17) $${\delta \omega }_{\text{in}}^{n}={\delta \omega }_{\text{ie}}^{n}+{\delta \omega }_{\text{en}}^{n}$$

(18) $${\delta \omega }_{\text{ie}}^{n}=\left[\begin{array}{c} -\omega_{\text{ie}}\sin\varphi \delta \varphi \\ 0\\ -\omega_{\text{ie}}\text{cos}\varphi \delta \varphi \end{array}\right]$$

(19) $${\delta \omega }_{\text{en}}^{n}=\left[\begin{array}{c} \frac{1}{R_{N}+h}{\delta V}_{E}-\frac{V_{E}}{{\left(R_{N}+h\right)}^{2}}\delta h\\ -\frac{1}{R_{M}+h}{\delta V}_{N}+\frac{V_{N}}{{\left(R_{M}+h\right)}^{2}}\delta h\\ -\frac{\text{tan}\varphi }{R_{N}+h}{\delta V}_{E}-\frac{V_{E}\sec^{2}\varphi }{R_{N}+h}\delta \varphi +\frac{V_{E}\text{tan}\varphi }{{\left(R_{N}+h\right)}^{2}}\delta h \end{array}\right]$$

where ${\delta V}_{E}$ and${\;\delta V}_{N}$ are the errors of east velocity and north velocity, respectively; δφ and δh are the errors of latitude and height, respectively; and ${\delta \omega }_{\text{ie}}^{n}$ and ${\delta \omega }_{\text{en}}^{n}$ are the errors of $\omega_{\text{ie}}^{n}$ and $\omega_{\text{en}}^{n}$, respectively.

The conventional velocity update equation can be written as follows [21]:

(20) $${\dot{V}}^{n}=R_{b}^{n}f_{\text{ib}}^{b}-\left(2\omega_{\text{ie}}^{n}+\omega_{\text{en}}^{n}\right)V^{n}+g^{n}$$

where $V^{n}$ is the velocity in the navigation frame; $f_{\text{ib}}^{b}$ is the output of accelerometers; and $g^{n}$ is the gravity expressed in the navigation frame (the model of $g^{n}$ is proposed in [38]).

The velocity error model can be obtained by perturbing Equation (20), and the DCM can be approximated by the following expression:

(21) $${\tilde{R}}_{n}^{b}=R_{n}^{b}\left(I-\Xi \right)$$

where the designator ~ in the above variables represents variables with errors. Ξ is a skew symmetric matrix of attitude error and Ξ is given by:

(22) $$\Xi =\left[\begin{array}{ccc} 0 & -\delta \psi & \delta \theta \\ \delta \psi & 0 & -\delta \phi \\ -\delta \theta & \delta \phi & 0 \end{array}\right]$$

However, the above equation may introduce errors if large attitude errors exist, so the error matrix of DCM $\left({\Delta R}_{b}^{n}\right)$ is introduced for the derivation of velocity error equation. The DCM in Equation (21) can be replaced by:

(23) $${\tilde{R}}_{n}^{b}=R_{n}^{b}-R_{n}^{b}{\Delta R}_{b}^{n}$$

The velocity update equation with error interferences can be written as:

(24) $${\dot{\tilde{V}}}^{n}={\tilde{R}}_{b}^{n}{\tilde{f}}_{\text{ib}}^{b}-\left(2{\tilde{\omega }}_{\text{ie}}^{n}+{\tilde{\omega }}_{\text{en}}^{n}\right){\tilde{V}}^{n}+{\tilde{g}}^{n}$$

The velocity errors $\left({\delta \dot{V}}^{n}\right)$ can be obtained by the difference between Equations (20) and (24), ignoring the second-order error product $R_{n}^{b}{\Delta R}_{b}^{n}{\delta f}_{\text{ib}}^{b}$ and $\left(2{\delta \omega }_{\text{ie}}^{n}+{\delta \omega }_{\text{en}}^{n}\right){\delta V}^{n}$, yields:

(25) $${\delta \dot{V}}^{n}={\dot{\tilde{V}}}^{n}-{\dot{V}}^{n}={\text{ R}}_{b}^{n}{\delta f}_{\text{ib}}^{b}-R_{b}^{n}{\Delta R}_{b}^{n}f_{\text{ib}}^{b}-\left(2\omega_{\text{ie}}^{n}+\omega_{\text{en}}^{n}\right){\delta V}^{n}-\left(2{\delta \omega }_{\text{ie}}^{n}+{\delta \omega }_{\text{en}}^{n}\right)V^{n}+{\delta g}^{n}$$

The only unknown parameter in Equation (25) is ${\Delta R}_{b}^{n}$, which can be obtained in terms of Φ, and the relationship between ${\Delta R}_{b}^{n}$ and Φ can be expressed as [27]:

(26) $${\Delta R}_{b}^{n}=2\left[\begin{array}{ccc} -s_{n}^{2}({\left(\delta \theta \right)}^{2}+{\left(\delta \psi \right)}^{2}) & s_{n}(1+c_{n})\delta \psi +s_{n}^{2}\delta \phi \delta \theta & -s_{n}(1+c_{n})\delta \theta +s_{n}^{2}\delta \phi \delta \psi \\ -s_{n}(1+c_{n})\delta \psi +s_{n}^{2}\delta \phi \delta \theta & -s_{n}^{2}({\left(\delta \phi \right)}^{2}+{\left(\delta \psi \right)}^{2}) & s_{n}(1+c_{n})\delta \phi +s_{n}^{2}\delta \theta \delta \psi \\ s_{n}(1+c_{n})\delta \theta +s_{n}^{2}\delta \phi \delta \psi & -s_{n}(1+c_{n})\delta \phi +s_{n}^{2}\delta \theta \delta \psi & -s_{n}^{2}({\left(\delta \phi \right)}^{2}+{\left(\delta \theta \right)}^{2}) \end{array}\right]$$

The velocity error equation is obtained by inserting Equation (26) into Equation (25). There is no relationship between the model position error $\left(\delta \dot{P}\right)$ and the attitude, so the position error model is still linear as [39]:

(27) $$\delta \dot{P}=\left[\begin{array}{c} \frac{{\delta V}_{N}}{R_{M}+h}-\frac{V_{N}}{{\left(R_{M}+h\right)}^{2}}\delta h\\ \frac{{\delta V}_{E}}{\left(R_{N}+h\right)\text{cos}\varphi }-\frac{V_{E}\delta h}{{\left(R_{N}+h\right)}^{2}\text{cos}\varphi }-\frac{V_{E}\delta \varphi }{\left(R_{N}+h\right)\cos^{2}\varphi }\\ -{\delta V}_{D} \end{array}\right]$$

where ${\delta V}_{D}$ is the error of the down velocity.

The nonlinear error model of the MGWD system can be achieved through Equations (7), (25) and (27). The observation equation is the linear ZUPT equation, which is expressed as follows:

(28) $$Z_{k}={\text{HX}}_{k}+v_{k}$$

where $Z_{k}$ is the difference between the estimate state $\left(V_{N},\;V_{E},\;V_{D},\;\varphi ,\;\lambda ,\text{ h}\right)$ and the zero velocity and zero position ($V_{N,0},\;V_{E,0},\;V_{D,0},\;\varphi_{0},\;\lambda_{0},\;h_{0}$):

(29) $$Z_{k}=\left[\begin{array}{c} \begin{array}{c} V_{N}-V_{N,0}\\ V_{E}-V_{E,0}\\ V_{D}-V_{D,0} \end{array}\\ \begin{array}{c} \varphi -\varphi_{0}\\ \lambda -\lambda_{0}\\ h-h_{0} \end{array} \end{array}\right]$$

(30) $$H=\left[\begin{array}{ccc} H_{11} & H_{12} & H_{13}\\ H_{21} & H_{22} & H_{23} \end{array}\right]$$

where λ is the longitude and $V_{N,0},\;V_{E,0},\;V_{D,0},\;\varphi_{0},\;\lambda_{0},\text{ and }h_{0}$ represent the zero velocity and zero position. $H_{12},\;H_{13},\;H_{21},\text{ and }H_{23}$ are a 3 × 3 zero matrix; $H_{11},\;H_{22}$ are a 3 × 3 identity matrix.

## 3. Recursive Bayesian Estimation

**Figure 1:**
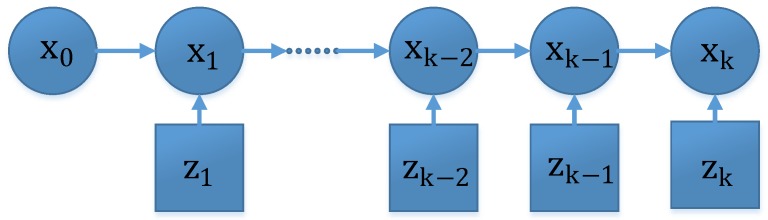
Recursive Bayesian estimation model. The first horizontal row represents the states of the system, which propagate in terms of Equation (31), and the column represents the measurement process, which is formulated by Equation (32). $x_{0},\;x_{1},\;x_{k-2},\;x_{k-1},\;x_{k}$ are the system states from time 0 to time k and $z_{1},\;z_{k-2},\;z_{k-1},\;z_{k}$ are the measurements from time 1 to time k.

In this section, we briefly review the theory of the recursive Bayesian estimation. The system is generally modeled as an SSM, which includes a system equation and an observation equation in the discrete time domain; the SSM can be written as follows:

(31) $$x_{k}=f_{k-1}\left(x_{k-1},\varpi_{k-1}\right)$$

(32) $$z_{k}=h_{k}\left(x_{k},{\text{ v}}_{k}\right)$$

where $f_{k-1}:$ ${\mathbb{R}}^{n}\times {\mathbb{R}}^{m}\to {\mathbb{R}}^{n}$ is the system nonlinear function; $\varpi_{k-1}\in {\mathbb{R}}^{m}$ is a zero mean, white noise with known pdf $p\left(\varpi_{k-1}\right)$; $x_{k}$ and $x_{k-1}$ are the system state vectors at present time k and previous moment k-1; $z_{k}\in {\mathbb{R}}^{n}$ is the measurement from external reference; $h_{k}:$ ${\mathbb{R}}^{n}\times {\mathbb{R}}^{r}\to {\mathbb{R}}^{p}$ is the measurement function; and $v_{k}\in {\mathbb{R}}^{r}$ is a zero mean, white noise with known pdf $p\left(v_{k}\right)$. Figure 1 illustrates the recursive Bayesian estimation model in graphical form [40,41].

A more general system model (Markov model) and observation model corresponding to the Equations (31) and (32) can be given by the following expressions:

(33) $$x_{k}\sim p\left(x_{k}|x_{k-1}\right)$$

(34) $$z_{k}\sim p(z_{k}|x_{k})$$

The definition of SSM in Equations (33) and (34) are based on the prior function and likelihood function. Both models are equivalent, but the general model is more convenient for the theoretical derivation.

The assumptions of recursive Bayesian estimation are that (1) the state vector $x_{k}$ is a hidden Markov process with initial distribution $p\left(x_{0}\right)$ and $x_{k}$ is independent of the other state vectors except x_k−1_, *i.e.*, the conditional pdf $p\left(x_{k}|x_{0:k-1}\right)=p\left(x_{k}|x_{k-1}\right)$; (2) the measurement process $z_{k}$ only depends upon $x_{k}$, *i.e.*, the conditional pdf $p\left(z_{k}{|x}_{k},{\text{ z}}_{1:k-1}\right)=p\left(z_{k}|x_{k}\right)$.

To sum up, the evaluation of the Bayesian estimation is given by the following steps.

(1) Prediction: evaluating the prior pdf $p\left(x_{k}{|z}_{1:k-1}\right)$ by the knowledge of the observation before time k in the light of the Chapman–Kolmogorov equation [42]:
  (35) $$p\left(x_{k}{|z}_{1:k-1}\right)=\int^{\text{​}}p\left(x_{k}{|x}_{k-1}\right)p(x_{k-1}|z_{1:k-1}){\text{dx}}_{k-1}$$
  where the pdf of initial state $p(x_{0}|z_{0})$ can be assigned by $p\left(x_{0}{|z}_{0}\right)=p\left(x_{0}\right)$. $p\left(x_{k}{|x}_{k-1}\right)$ is the prior distribution, which is given by [43]:
  (36) $$p\left(x_{k}{|x}_{k-1}\right)=\int^{\text{​}}\delta \left(x_{k}-f_{k-1}\left(x_{k-1},w_{k-1}\right)\right)p\left(\varpi_{k-1}\right)d\varpi_{k-1}$$
  where δ(⋅) is the Dirac delta function.
(2) Updating: the posterior pdf $p\left(x_{k}{|z}_{1:k}\right)$ can be updated with the new measurement $z_{k}$ at time k in terms of the Bayes rule as follows [44]:
  (37) $$p\left(x_{k}{|z}_{1:k}\right)=\frac{p(z_{k}|x_{k})p\left(x_{k}{|z}_{1:k-1}\right)}{p\left(z_{k}{|z}_{1:k-1}\right)}$$
  where $p\left(z_{k}{|x}_{k}\right)$ is the likelihood function, which is written by the following equation [43]:
  (38) $$p\left(z_{k}{|x}_{k}\right)=\int^{\text{​}}\delta \left(y_{k}-h_{k}\left(x_{k},\;v_{k}\right)\right)p\left(v_{k}\right){\text{dv}}_{k}$$
(3) State estimation: once the posterior is obtained, the estimation of state $\left({\hat{x}}_{k}\right)$ and the covariance matrix of estimation error ($P_{{\hat{x}}_{k}}$) are given by [45]:
  (39) $${\hat{x}}_{k}=E\left(x_{k}|z_{1:k}\right)=\int^{\text{​}}x_{k}p\left(x_{k}|z_{1:k}\right){\text{dx}}_{k}$$
  (40) $$P_{{\hat{x}}_{k}}=E\left({\tilde{x}}_{k}{\tilde{x}}_{k}^{T}\right)=\int^{\text{​}}{\tilde{x}}_{k}{\tilde{x}}_{k}^{T}p\left(x_{k}|z_{1:k}\right){\text{dx}}_{k}$$
  where ${\tilde{x}}_{k}=x_{k}-{\hat{x}}_{k}$ is the estimation error and E(⋅) is the expectation of the random variables.

## 4. PF Algorithm

Because the function $p\left(z_{k}{|z}_{1:k-1}\right)$ in Equation (37) cannot typically be computed analytically, sequential Monte Carlo (SMC) approximation, the key idea of the PF, was proposed for the hidden states estimation of the dynamic systems [46,47]. The PF uses a finite set of weighted particles or samples to reconstruct the posterior pdf $p\left(x_{0:k}{|z}_{1:k}\right)$ of the state vectors regardless of the nonlinear or non-Gaussian systems [48,49]. The implementation of the PF can be summarized in three main steps: sequential importance sampling (SIS), resampling, and roughening. The SIS plays a fundamental rule in the iteration of the PF since searching proper importance function decides the success of PF. Resampling aims at alleviating the particles’ or samples’ degeneracy and the roughing approach can be utilized to avoid the particles’ or samples’ impoverishment.

### 4.1. SIS Algorithm

The SIS algorithm steps from the literature [50,51]. The basic idea of SIS is the selection of particles or samples with weights to represent the complicated high dimensional distributions [52]. The posterior pdf $p(x_{0:k}|z_{1:k})$ is approximated by the random particles or samples and weights $\left\{x_{0:k}^{i},w_{k}^{\left(i\right)}|i=1,2,\cdots N\right\}$ at time k as:

(41) $$p(x_{0:k}|z_{1:k})=\overset{N}{\underset{i=1}{\sum }}w_{k}^{\left(i\right)}\delta \left(x_{0:k}-x_{0:k}^{i}\right)$$

where N is the number of particles and $x_{0:k}$ is the system state.

The first task to implement the SIS is the selection of the importance function $\pi \left(x_{0:k}{|z}_{1:k}\right),$ which is proportional of the posterior pdf $p(x_{0:k}|z_{1:k})$ and is easy to draw samples compared with $\text{ p}(x_{0:k}|z_{1:k})$. The weights can be updated in terms of the following equation:

(42) $$w_{k}^{\left(i\right)}=\frac{p\left(x_{0:k}{|z}_{1:k}\right)p\left(z_{1:k}\right)}{\pi \left(x_{0:k}{|z}_{1:k}\right)}\propto \frac{p\left(x_{0:k}{|z}_{1:k}\right)}{\pi \left(x_{0:k}{|z}_{1:k}\right)}$$

Generally, the importance function is selected such that [53]:

(43) $$\pi \left(x_{0:k}{|z}_{1:k}\right)=\pi (x_{k}{|x}_{k-1},z_{k})\pi \left(x_{0:k-1}{|z}_{1:k}\right)$$

Note that the Equation (41) can be factored as following equation according to the Bayesian rule [54]:

(44) $$p\left(x_{0:k}{|z}_{1:k}\right)\propto p\left(z_{k}|x_{0:k}\right)p\left(x_{k}|x_{k-1}\right)p\left(x_{0:k-1}{|z}_{1:k-1}\right)$$

Therefore, the weights can be updated by substituting Equations (43) and (44) into Equation (42), yielding:

(45) $$w_{k}^{\left(i\right)}\propto w_{k-1}^{\left(i\right)}\frac{p\left(z_{k}|x_{k}^{i}\right)p\left(x_{k}^{i}|x_{k-1}^{i}\right)}{\pi \left(x_{k}^{i}{|x}_{k-1}^{i},z_{k}\right)}$$

where $x_{k}^{i}$ is the variable obtained by the evaluation of ${\text{ x}}_{k-1}^{i}$ in the system model and $x_{k-1}^{i}$ is the samples drawn from the importance function $\pi \left(x_{k}^{i}{|x}_{k-1}^{i},z_{k}\right),$ which is related to the previous state $x_{k-1}^{i}$ and current observation $z_{k}$. In the application of the MGWD system, the estimation of the current state is more important than the previous states, so Equation (41) can be rewritten as:

(46) $$p(x_{k}|z_{1:k})=\overset{N}{\underset{i=1}{\sum }}w_{k}^{\left(i\right)}\delta \left(x_{k}-x_{k}^{i}\right)$$

As indicated in Equations (45) and (46), the posterior pdf depends on the likelihood function, the priori pdf, and the importance function. Since the likelihood function and the priori pdf can be obtained in terms of the SSM, the choice of importance function is essential for PF. The optimal function, the likelihood function, and the priori function are the components of the importance function [54].

The optimal function $p\left(x_{k}^{i}{|x}_{k-1}^{i},z_{k}\right)$ was proposed as the importance function in [41]:

(47) $$p\left(x_{k}^{i}{|x}_{k-1}^{i},z_{k}\right)=p\left(x_{k}|x_{k-1}^{i}\right)$$

Substituting Equation (47) into Equation (45) yields:

(48) $$w_{k}^{\left(i\right)}\propto w_{k-1}^{\left(i\right)}p\left(z_{k}|x_{k-1}^{i}\right)$$

The drawbacks of using optimal importance function are: (1) it is difficult to draw samples from the optimal function $p\left(x_{k}^{i}{|x}_{k-1}^{i},z_{k}\right)$; (2) it needs the integration calculation to update the weights. The optimal function can be factorized as the following format based on the Bayesian rule:

(49) $$p\left(x_{k}^{i}{|x}_{k-1}^{i},z_{k}\right)=\frac{p\left(z_{k}|x_{k}^{i}\right)p\left(x_{k}^{i}|x_{k-1}^{i}\right)}{p\left(z_{k}|x_{k-1}^{i}\right)}$$

As indicated in Equation (49), $p\left(x_{k}^{i}{|x}_{k-1}^{i},z_{k}\right)\propto p\left(z_{k}|x_{k}^{i}\right)$ if the likelihood function $p\left(z_{k}|x_{k}^{i}\right)$ is more noisy and it is integral in $x_{k}^{i}$ [55]. In this case, the importance function can be presented by the likelihood function, namely:

(50) $$\pi \left(x_{k}^{i}{|x}_{k-1}^{i},z_{k}\right)=p\left(z_{k}|x_{k}^{i}\right)$$

Then, the weight in Equation (45) can be updated by the following equation:

(51) $$w_{k}^{\left(i\right)}\propto w_{k-1}^{\left(i\right)}p\left(x_{k}^{i}|x_{k-1}^{i}\right)$$

However, the likelihood function as the importance function can only be used in the high signal-to-noise ratio (SNR) case. In this paper, the priori function is selected as the importance function because of the tradeoff between computational complexity and the feasibility of implementation. The importance function is written by:

(52) $$\pi \left(x_{k}^{i}{|x}_{k-1}^{i},z_{k}\right)=p(x_{k}^{i}|x_{k-1}^{i})$$

Then, the weights can be updated by:

(53) $$w_{k}^{\left(i\right)}\propto w_{k-1}^{\left(i\right)}p\left(z_{k}|x_{k}^{i}\right)$$

The above equation shows that the weights only depend on the previous weights and the likelihood function, which are available from the knowledge of the observation equation and the pdf of the observation noise. In this application, we assume that the measurement noise of the MGWD system is additive, Gaussian white noise, so the likelihood function can be evaluated in a simple way [56]:

(54) $$P\left(z_{k}│x_{k}^{\left(i\right)}\right)\approx \frac{1}{2\pi {\left|R\right|}^{1/2}}\text{ exp}\left(-{\left(z_{k}-h\left(x_{k}^{i}\right)\right)}^{T}R^{-1}\left(z_{k}-h\left(x_{k}^{i}\right)\right)\right)$$

where R is the covariance matrix of measurement noise $v_{k}$.

The normalization of the particle weights are performed to guarantee the sum of the particle weights equal to 1, and the normalization of the weights can be expressed as follows:

(55) $$w_{k}^{\left(i\right)}=\frac{w_{k}^{\left(i\right)}}{\sum_{i=1}^{N}w_{k}^{\left(i\right)}}$$

Under the premise that the initial pdf $p(x_{0}|z_{0})$ is known, drawing particles ${\text{ x}}_{0}^{i}\sim p(x_{0}|z_{0}),\text{ where i}=1,2,N$, as:

(56) $$x_{k-1}^{i}=f_{k-1}\left(x_{0}^{i},\varpi_{0}^{i}\right)$$

where $x_{0}^{i}$ is the initial particles and $\varpi_{0}^{i}$ is the noise vector which is drawn from the pdf of system noise $p\left(\varpi_{k-1}\right)$. The initial weights are given by:

(57) $$w_{0}^{i}=p\left(z_{0}|x_{0}^{i}\right)$$

The SIS algorithm can only be implemented in special cases because the variance of the importance weights only increase over time; eventually, they give rise to particle degeneracy. Particle degeneracy means that the updating particles are useless for the approximation of a posterior pdf. In order to solve the particles degeneracy problem, a resampling method is described in next section.

### 4.2. Resampling Algorithm

**Figure 2:**
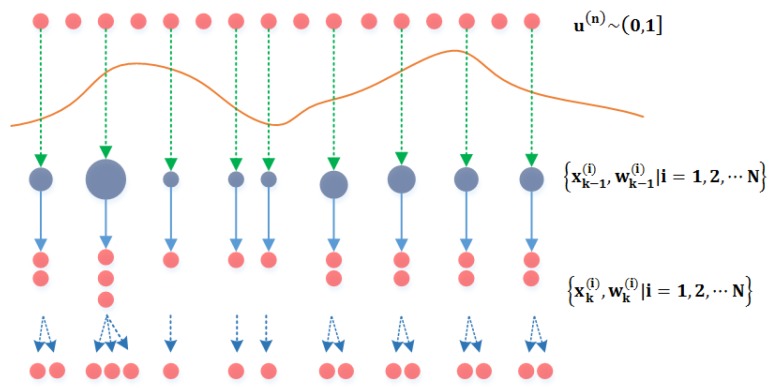
Principle of resampling.

The PF would suffer from the particle degeneracy without a resampling algorithm. Figure 2 shows the principle of the particle resampling.

The key of the resampling algorithm is that particles with large weights are replicated and particles with small weights are discarded [57]. The effective sample size $N_{\text{eff}}$ is an indicator of the degree of the particles’ degeneracy and a metric of employing the particles’ resampling. $N_{\text{eff}}$ is provided by the following equation [58]:

(58) $$N_{\text{eff}}=\frac{N}{1+N^{2}\text{var}\left(w_{k}^{\left(i\right)}\right)}\approx \frac{1}{\sum_{i=1}^{N}{\left(w_{k}^{\left(i\right)}\right)}^{2}}$$

where $var\left(w_{k}^{\left(i\right)}\right)$ is the variation of the weight.

The particles should be resampled if $N_{\text{eff}}$ is less than resample threshold $\left(N_{\text{th}}\right)$, which equals 4N/5 in this paper. Equation (58) shows that $N_{\text{eff}}$ approaches 1 if the particles are severely degenerated; on the other hand, $N_{\text{eff}}$ approaches N if the particles have equal weight (1/N). The number of the resampled particles is not necessarily the same as the propagated particles N; however, the number of particles can be the same for computational convenience. Therefore, the total resampling particles are unchanged, still N, so the resampled particles have the same weight (1/N).

There are several ways to implement the resampling algorithm such as systematic resampling [59], multinomial resampling [60], residual resampling [61], *etc.* The resampling strategy in PF is sampling with replacement, that is, the particles drawn from the box will be replaced after the decimation. In this paper, the multinomial resampling approach is selected to alleviate particles’ degeneracy. The multinomial resampling can be considered as a series of Bernoulli trials. N random samples $u^{\left(n\right)},\text{ n}=1,2,\cdots N$ are generated from the uniform distribution over (0,1] and the probabilities of resampling the particles is the same as $u^{\left(n\right)}$ when the following inequality is satisfied:

(59) $$\overset{N-1}{\underset{i=1}{\sum }}w_{k}^{\left(i\right)}<u^{\left(n\right)}\leq \overset{N}{\underset{i=1}{\sum }}w_{k}^{\left(i\right)}$$

Then, the nth particle $x_{k}^{\left(n\right)}$ with the nth weight is the selection of the new samples or particles $x_{k}^{\left(i\right)}$ of the resampling.

### 4.3. Roughing Strategy

The problem of resampling is that only a few particles are drawn, which is also referred to as samples impoverishment. Therefore, the particles lack diversities because the selection may include many repeated particles. In order to address the samples impoverishment problem, the random noise is added to the particles after resampling. The posterior particles $x_{k}^{\left(i\right)+}$ are written as:

(60) $$x_{k}^{\left(i\right)+}=x_{k}^{\left(i\right)}+\varrho_{k}^{\left(i\right)}$$

where $\varrho_{k}^{\left(i\right)}$ is a zero mean random noise.

At this point, the particles after the roughing step can be propagated for the update of the weights and the posterior pdf in the next time cycle. The first two moments of estimation derived from the particles can be approximated by the following equations:

(61) $${\hat{x}}_{k}=E\left[x_{k}\right]\approx \overset{N}{\underset{i=1}{\sum }}w_{k}^{i}\left(x_{k}^{\left(i\right)+}\right)$$

(62) $$P_{{\hat{x}}_{k}}=E\left[\left(x_{k}-{\hat{x}}_{k}\right){\left(x_{k}-{\hat{x}}_{k}\right)}^{T}\right]\approx \overset{N}{\underset{i=1}{\sum }}w_{k}^{i}\left(x_{k}^{\left(i\right)+}\right){\left(x_{k}^{\left(i\right)+}\right)}^{T}$$

## 5. Results and Discussion

The objectives of the designed experiments are to demonstrate that: (1) the performance of the PF with NEM or NNEM is better than KF with LEM under small-angle attitude error condition (Experiment 1); (2) PF with NNEM can provide more accurate states estimation for the MGWD system with large-angle attitude error (Experiment 2). The configuration of the MGWD device and the details of the experimental data collection are briefly described in the first part of Section 5.

### 5.1. MGWD Device for Validation of Experimental Results

**Figure 3:**
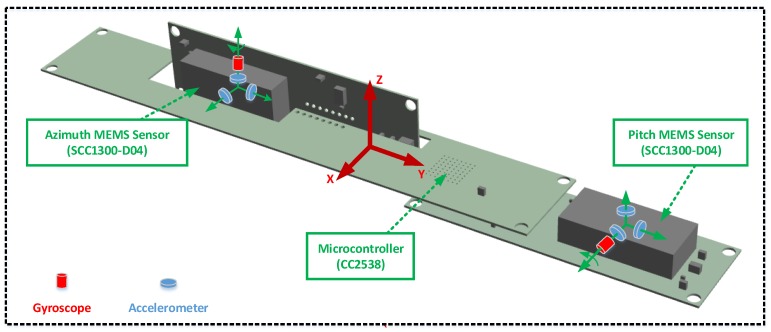
Configuration of the MGWD device. Each MEMS sensor is composed of a single-axis gyroscope and three-axis accelerometers. The body frame is the right (X-axis), forward (Y-axis) and up (Z-axis) direction.

The MGWD device consists of the azimuth MEMS sensor, the pitch MEMS sensor, and the microcontroller. Figure 3 shows the configuration of the MGWD device. Azimuth MEMS sensor provides the angular rate of the drill bit along the Z-axis while the angular rate along the X-axis is obtained from the pitch MEMS sensor. For implementation of the pure INS algorithm, the angular rate along the Y-axis is evaluated by the specific force obtained from the pitch MEMS sensor [12].

The MEMS sensor (SCC1300-D04, Murata Manufacturing Co., Ltd, Kyoto, Japan) is a combined gyroscope and accelerometer component and the sensor is characterized by low cost, small diameter, and high performance. The microcontroller (CC2538, Texas Instruments, Dallas, TX, USA) is a high-speed System-on-Chip (SoC) combined with ARM cortex-M3 processor. Table 1 and Table 2 provide the technical specification of the MEMS sensor and the microcontroller, respectively.

The types of data communication between the MEMS sensor and microcontroller is the serial peripheral interfaces (SPI) interface with 115,200 bit/s data transmission rate. The data collection software evaluates the navigation solutions with angular rate and specific force received from MGWD device through the universal serial bus (USB) with 20 Hz sample rate.

**Figure 4:**
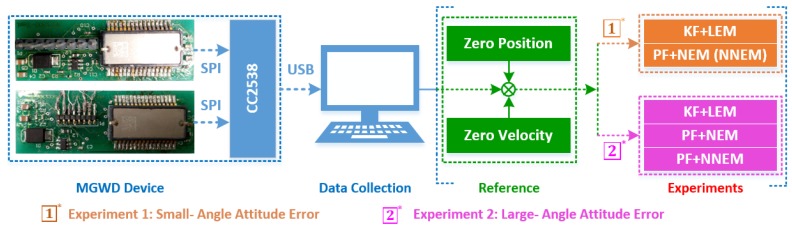
Process of data collection and contents of experiments. The MEMS sensors sense the angular rate and specific force of the drill bit and then transfer the data to the computer through a USB interface for evaluation of navigation solutions. The proposed algorithm would be verified by two groups of designed experiments with the reference of zero velocity and zero position. The design rule of experiments is based on the initial attitude error.

Figure 4 shows the process of the data collection and the contents of the experiments. The computer continuously reads the angular rate and the specific force of the drill bit from the static MGWD device. Therefore, the zero velocity and zero position are readily available as the reference of experiments, designed in terms of the size of attitude error.

**Figure 5:**
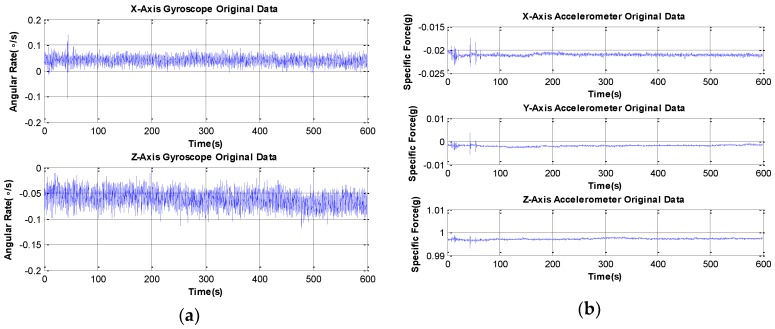
Original data obtained from experiments using the MGWD device. (**a**) Original data from two-axis gyroscopes; (**b**) original data from three-axis accelerometers. The length of experimental data is 600 s in quasi-stationary conditions.

In the experiment, we collect original data from the static MGWD device for the period of 10 min to demonstrate the performance of the PF and the accuracy of the proposed NNEM. Figure 5 shows the original data from two-axis gyroscopes and three-axis accelerometers. The outputs of gyroscopes include the noise and the angular rate of the Earth projected onto the X-axis and Z-axis of the MGWD device. The X-axis and the Y-axis outputs of accelerometers are only the noise since the device is under static condition while the Z-axis output of accelerometer consists of the noise and gravity.

### 5.2. Experiment 1: Comparison of KF with LEM and PF with NEM under Small-Angle Attitude Error Condition

The aim of Experiment 1 is to confirm that the performance of the PF with NEM is better than the performance of KF with LEM under small-angle attitude error conditions. The assumption of KF is that all the states are Gaussian and the SSM is linear. However, the complicated drilling environments introduce such strong interference to the MGWD device that the linear SSM and Gaussian states are impossible.

The first 50 s are utilized for initial alignment; the results of the initial alignment for roll, pitch, and azimuth are −0.022108°, 0.180922°, and −10.351435°, which are also set as the initial attitude in this experiment. Therefore, the initial attitude error is so small that the DCM can be approximated by the attitude. The rest of parameters related to the KF and PF are given as follows:

- Initial covariance matrix of system noise: R = diag([0.1,0.1, 0.1,0.1, 0.1, 0.1]);
- Initial covariance matrix of observation noise: Q = diag([0.1, 0.1, 0.1, 0.1, 0.1, 0.1, 0.1, 0.1, 0.1]);
- Initial position: latitude 126.6879°; longitude 45.7776°; height 124 m;
- Initial velocity: 0.

**Figure 6:**
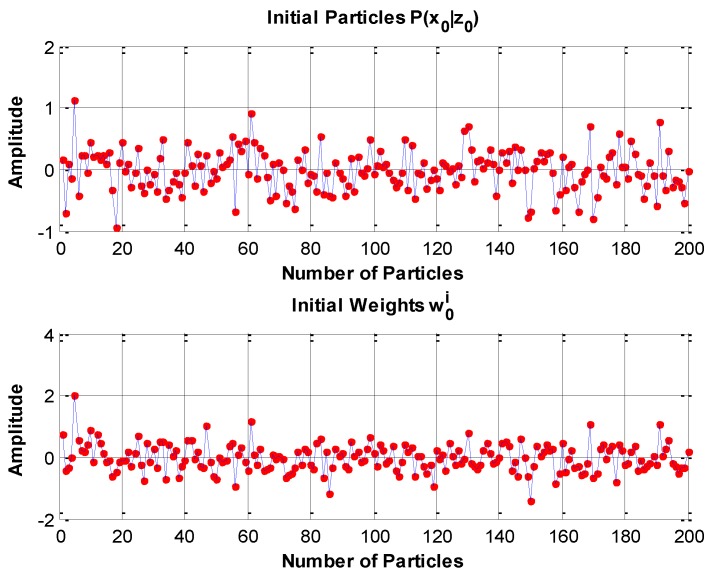
Initial particles $p\left(x_{0}|z_{0}\right)$ and initial weights $w_{0}^{i}$ for the PF. The initial particles and weights of attitude errors (δϕ,δθ,δψ), velocity errors $\left({\delta V}_{N},\;{\delta V}_{E},\;{\delta V}_{D}\right)$, and position errors (δφ, δλ, δh) are identical because the initial parameters (initial states and initial estimation errors) to evaluate the initial particles and initial weights are the same.

Figure 6 illustrates the initial particles and initial weights.

**Figure 7:**
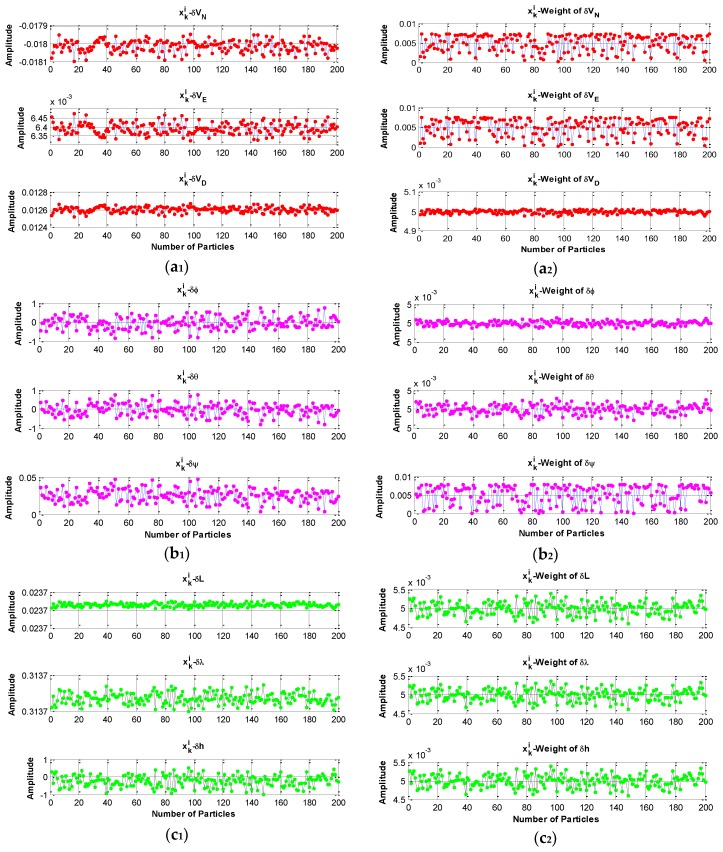
Particles or samples, along with the corresponding weights for evaluation of the posterior function. (**a_1_**) and (**a_2_**) are particles of velocity errors and the corresponding weights; (**b_1_**) and (**b_2_**) are the particles of attitude errors and the corresponding weights; (**c_1_**) and (**c_2_**) are the particles of positon errors and the corresponding weights. The priori pdf is the importance density function. The designator δL, the same as δφ, is the latitude error.

Figure 7 a_1_, a_2_, b_1_, b_2_, c_1_, c_2_ show the particles of the state errors together with the corresponding weights drawn from the priori pdf $\left\{p\left(x_{k}^{i}|x_{k-1}^{i}\right)\right\}$ at time k. The posterior pdf $\left\{p\left(x_{k}|z_{k}\right)\right\}$ can be evaluated by the Equation (46) after the particles and the corresponding weights are obtained. The number of the particles and weights for each state are 200. The particles and weights are updated at every time circle; meanwhile, the posterior pdf is updated.

**Figure 8:**
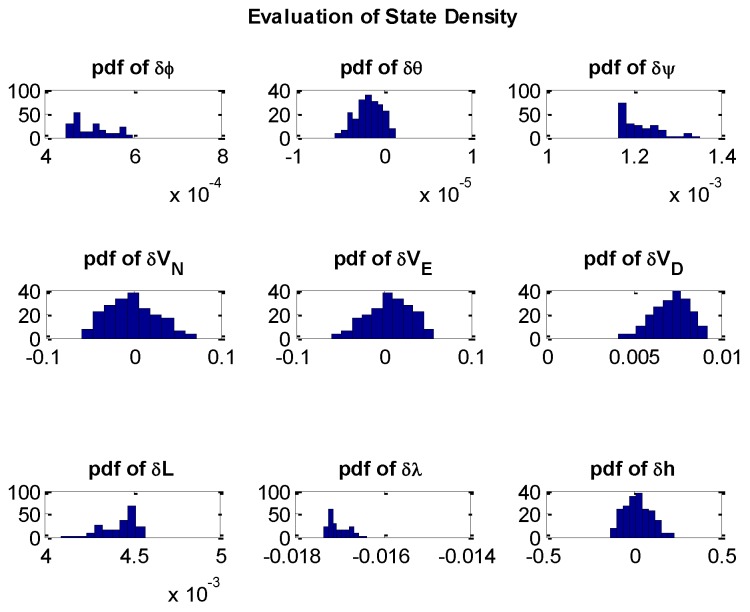
Evaluation of state density.

Figure 8 presents the evaluation of state density in terms of the updated pdf; the results illustrates that the pdf of states are partially Gaussian. Therefore, the KF is suboptimal since the assumption is that all states are Gaussian in KF while the PF would not be limited by the non-Gaussian assumption.

**Figure 9:**
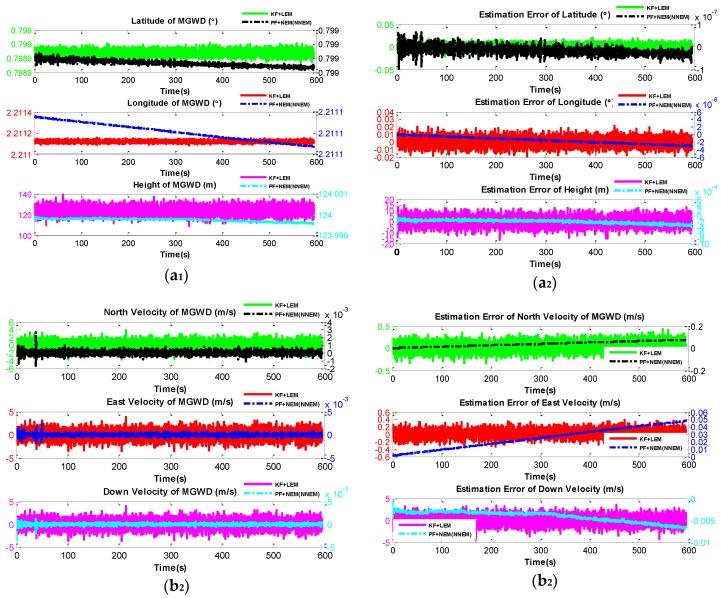

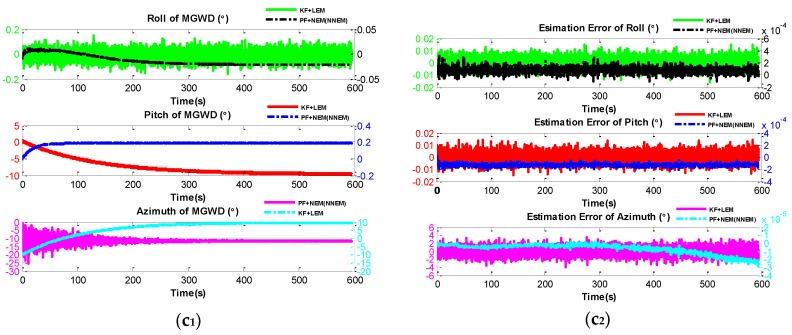
Comparison of the performance of KF with LEM and PF with NEM or NNEM under small-angle attitude errors conditions. (**a_1_**) and (**a_2_**) are the estimated attitude and the corresponding estimation error; (**b_1_**) and (**b_2_**) are the estimated velocity and the corresponding estimation error; (**c_1_**) and (**c_2_**) are the estimated position and the corresponding estimation error. Plotting the figure uses the double Y-axis plotting approach because the results are in different orders of magnitude.

Figure 9 gives a performance comparison of the KF with LEM and PF with NEM or NNEM under small-angle attitude error. Figure 9 a_1_, a_2_ show that both the latitude and the longitude estimated by KF and PF converge to 45.7776° and 126.6879°, which are the same as the reference values (initial latitude and initial longitude). However, the height estimated by KF is 132.0782 m while the height estimated by PF is 123.9999 m, which is almost the same as the reference height (124 m). Therefore, the NEM or NNEM can restrain the errors of height effectively under small-angle attitude error conditions.

In Figure 9 b_1_, b_2_ the north velocity, east velocity, and down velocity estimated by PF are close to zero (0.0002 m/s, 0.0001 m/s, and 0.0001 m/s) while the errors of north velocity, east velocity, and down velocity estimated by KF are up to 0.3702 m/s, 0.2839 m/s, and 2.2953 m/s respectively. Therefore, the PF with NEM or NNEM gives better performance than KF with LEM does for the velocity estimation under small-angle attitude error.

In Figure 9 c_1_, c_2_ the roll, pitch, and azimuth of MGWD system estimated by PF with NEM or NNEM largely converge to −0.022125°, 0.180922°, and −10.353632°, respectively, which are the same as the reference points provided by the initial alignment, while the results of pitch and azimuth estimated by KF deviate from the reference values by about 8° and 10°, respectively. Therefore, the PF with NEM or NNEM can restrain the pitch and azimuth error effectively.

### 5.3. Experiment 2: Validation of the Performance of NNEM with Large-Angle Attitude Error

The objective of Experiment 2 is the validation of the performance of NNEM with large-angle attitude error. The experimental data used in this section are the same as those in Experiment 1 and the initial parameter settings are also the same as in experiment 1 apart from the initial attitude. The assumption of this experiment is that the initial alignment fails to provide an accurate initial attitude. We set the initial roll, pitch, and azimuth as 0°. The experiment is practical since the initial attitude is generally difficult to obtain in the application of downhole drilling.

**Figure 10:**
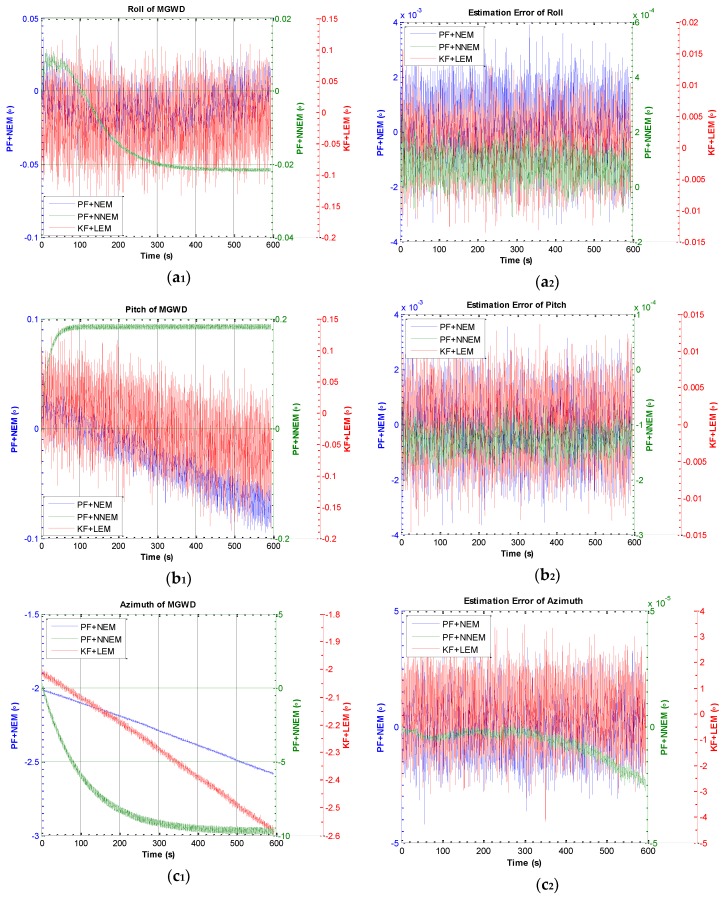
Results of attitude and estimation error of attitude by KF with LEM, PF with NEM, and PF with NNEM under large-angle attitude error conditions. (**a_1_**) and (**a_2_**) are the estimation of roll and estimation error of roll; (**b_1_**) and (**b_2_**) are the estimation of pitch and estimation error of pitch; (**c_1_**) and (**c_2_**) are the estimation of azimuth and estimation error of azimuth. Plotting the figure uses the triple Y-axis plotting approach because the results are in different orders of magnitude.

Figure 10 a_1_, a_2_, b_1_, b_2_, c_1_, c_2_ show the estimation of roll, pitch, and azimuth of the MGWD system and the corresponding estimation errors. The roll, pitch, and azimuth converge to −0.021976°, 0.184392°, and −9.893302°, respectively, which are close to the reference values for initial alignment. The selection of initial attitude has little influence on the convergence of the attitude estimation by PF with NNEM since the roll converges to the reference value at around 320 s, the pitch converges to the reference value at around 180 s, and the azimuth converges to the reference value at around 360 s, as illustrated in Figure 10 a_1_, b_1_, and c_1_. Figure 10 a_2_, b_2_, c_2_ show that the error of roll and error of pitch estimated by PF with both NNEM and NEM are less than 0.0001° and 0.001°. The error of azimuth estimated by PF with NEM is 2.459532° while the estimation error of azimuth with NNEM is $2.5\times 10^{-5}{}^{\circ}$. In addition, the accurate solutions of roll and pitch presented in Figure 10 demonstrate that the error of roll and error of pitch couple with the accuracy of east velocity and north velocity. Therefore, the PF with NNEM can greatly restrain the azimuth error under large-angle latitude error conditions.

**Figure 11:**
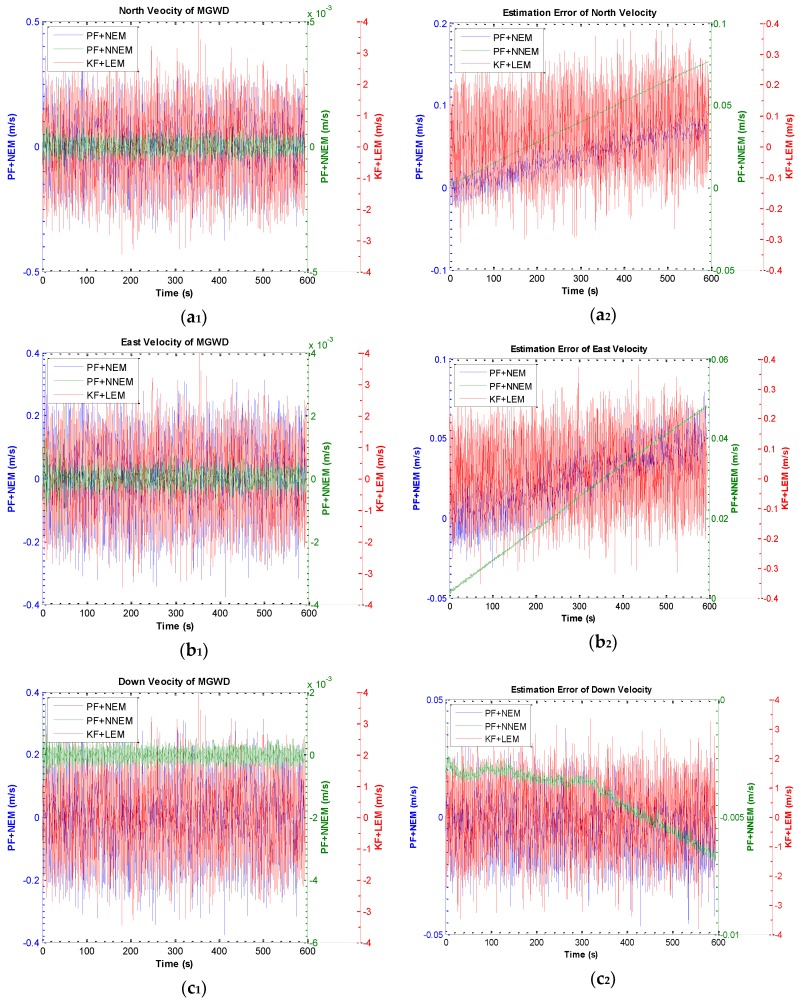
Results of estimation of velocity and estimation error of velocity by KF with LEM, PF with NEM, and PF with NNEM under large-angle attitude error conditions. (**a_1_**) and (**a_2_**) are the estimation of north velocity and estimation error of north velocity; (**b_1_**) and (**b_2_**) are the estimation of east velocity and estimation error of east velocity; (**c_1_**) and (**c_2_**) are the estimation of down velocity and estimation error of down velocity.

Figure 11 a_1_, a_2_, b_1_, b_2_, c_1_, c_2_ illustrate the results of the north velocity, east velocity, and down velocity and the corresponding estimation errors of the MGWD system. The velocity estimated by PF, regardless of NEM or NNEM, apparently converges to zero velocity while the velocity error estimated by PF with NNEM is approximately 10 times smaller than KF with LEM. Therefore, the velocity errors estimated by PF with NNEM can be reduced effectively.

**Figure 12:**
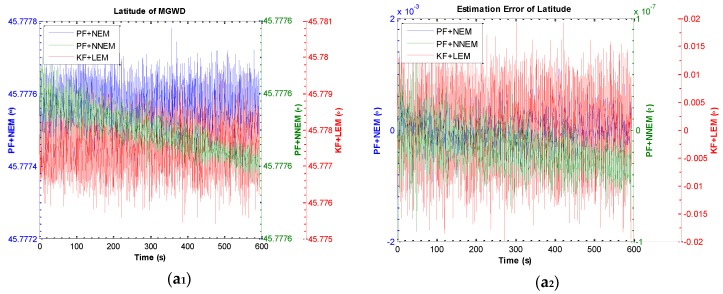

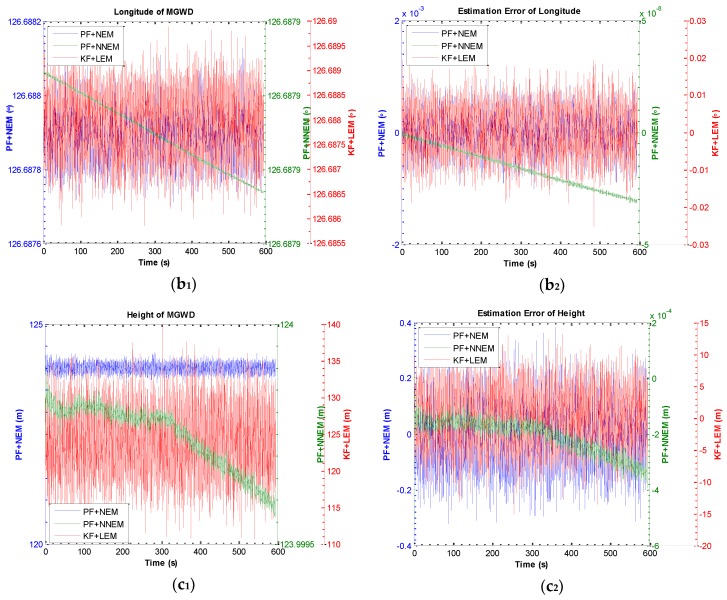
Results of estimation of position and estimation error of position by KF with LEM, PF with NEM, and PF with NNEM under large-angle attitude error condition. (**a_1_**) and (**a_2_**) are the estimation of latitude and estimation error of latitude; (**b_1_**) and (**b_2_**) are the estimation of longitude and estimation error of longitude; (**c_1_**) and (**c_2_**) are the estimation of height and estimation error of height.

Figure 12 a_1_, a_2_, b_1_, b_2_, c_1_, c_2_ describe the estimation of latitude, longitude, and height of the MGWD system and the corresponding estimation errors. The latitude and longitude estimated by KF with LEM, PF with NEM, and PF with NNEM are almost close to the reference values 45.7776° for latitude, 126.6879° for longitude. The height estimated by PF with NNEM is 123.9996 m (reference height: 124 m) while the error of height estimated by KF with LEM is about 5 m and the error of height estimated by PF with NEM is approximately 3 m. The results demonstrate that the PF with NNEM can constrain the error growth of height effectively. Table 3 lists the results of Experiment 2.

## 6. Conclusions

This paper proposes a NNEM for MGWD system under large-angle attitude error conditions. The error of DCM as the intermediate variable is introduced for the derivation of NNEM, which can effectively eliminate the approximation of errors existing in the derivation of conventional NEM. The evaluation of the state density shows that the system states are partially Gaussian, so the KF is suboptimal in this application. PF can improve the estimation performance of nonlinear problems by approximating the posterior pdf with the particles and weights. The PF with NEM gives a better performance than KF with LEM for the estimation of height, velocity, pitch, and azimuth in Experiment 1. The NNEM of the MGWD system provides an accurate estimation of position, velocity, and attitude compared with PF with NEM and KF with LEM in Experiment 2.

## Figures and Tables

**Table 1:** Technical specifications for SCC1300-D04.

Parameters	Gyroscope	Parameters	Accelerometer
Offset Short Term Instability	<2.1 °/h	Offset Error	±70 mg
Angular Random Walk	0.86 °/$\sqrt{h}$	Linearity Error	±40 mg
Noise Density	0.02 (°/s/$\sqrt{h})$	Noise	5 ~ 7 mg
Temperature	−40 ~ +125 °C	Temperature	−40 ~ +125 °C

**Table 2:** Technical specifications for CC2538.

Parameters	Values	Parameters	Values
Processor	ARM Cortex-M3	Debugging	cJTAG and JTAG
Frequency	24 MHz	RF	2.4 GHz IEEE 802.15.4 Transceiver
Peripherals	USB/I2C/SSI/UART	Size	8 mm × 8 mm
Temperature	−40 ~ +125 °C	Voltage	2 V ~ 3.6 V

**Table 3:** Navigation solutions and corresponding errors estimated by PF with NNEM, PF with NEM, and KF with LEM in Experiment 2.

Parameters	PF + NNEM	PF + NEM	KF + LEM
φ	45.777664°	45.777613°	45.777935°
λ	126.687953°	126.687934°	126.688326°
h	123.999686 m	123.652484 m	120.945752 vm
$V_{N}$	0.002984 m/s	0.0197424 m/s	0.983472 m/s
$V_{E}$	−0.000019 m/s	−0.234530 m/s	1.342358 m/s
$V_{D}$	0.005637 m/s	0.287584 m/s	1.846332 m/s
ϕ	−0.021976°	−0.034748°	−0.053672°
θ	0.184392°	−0.068584°	−0.026478°
ψ	−9.893302°	−2.527547°	−2.599384°
δφ	−0.362394 × 10^−7^	−0.000014°	−0.000238°
δλ	−0.457395 × 10^−8^	−0.000106°	−0.000056°
δh	−0.000348 m	−0.196528 m	−8.872501 m
${\delta V}_{N}$	0.075922 m/s	0.128473 m/s	0.248382 m/s
${\delta V}_{E}$	0.048975 m/s	0.056483 m/s	0.287349 m/s
${\delta V}_{D}$	−0.00648 m/s	−0.039320 m/s	0.877265 m/s
δϕ	−0.000464°	0.003502°	0.004882°
δψ	0.000353°	0.004528°	0.005216°
δψ	−0.000025°	2.459532°	1.879347°
